# Editorial: The impact of lipid metabolism on cancer progression and metastasis

**DOI:** 10.3389/fmed.2025.1755140

**Published:** 2025-12-10

**Authors:** Duygu Aydemir, Miguel Martin-Perez, Yoshiaki Sunami

**Affiliations:** 1 Division of Medical Genetics, Department of Pediatric Basic Sciences, Institute of Child Health, Istanbul University, Istanbul, Türkiye; 2 Institute for Research in Biomedicine (IRB Barcelona), The Barcelona Institute of Science and Technology (BIST), Barcelona, Spain; 3 Department of Cell Biology, Physiology and Immunology, University of Barcelona, Barcelona, Spain; 4 Department of Visceral, Vascular and Endocrine Surgery, Martin-Luther-University Halle-Wittenberg, University Medical Center Halle, Halle, Germany

**Keywords:** cancer progression, immunometabolism, lipid metabolism, metastasis, prognostic biomarkers, tumor microenvironment

Recent advances in oncology, immunology, and metabolic medicine have increasingly positioned lipid metabolism as a central determinant of cancer initiation, progression, and therapeutic response. Across multiple domains, lipid metabolic pathways are now understood to intersect with endocrine regulation, immune cell function, and systemic metabolic homeostasis, thereby influencing tumor behavior at both cellular and organismal levels. This expanding body of evidence highlights lipid metabolism not as an isolated biochemical process, but as an integrative axis that coordinates metabolic, immunological, and endocrine signals within the tumor microenvironment and throughout the host. Such a multidimensional perspective underscores the importance of synthesizing cross-disciplinary findings to fully elucidate the mechanistic and translational relevance of lipid metabolic regulation in contemporary cancer research (Pascual and Benitah; Zhang et al.; Liu et al.; Song et al.; Kim et al.).

Pascual and Benitah's review underscored that lipid remodeling within the tumor microenvironment (TME) drives immune evasion and metastasis. The authors detailed how lipid-enriched milieus reprogram immune subsets, particularly T cells, macrophages, and neutrophils toward immunosuppressive phenotypes, emphasizing potential therapeutic targets such as CD36, PPARs, and cholesterol biosynthesis pathways. This work delineates how metabolic reprogramming within immune cells mirrors tumor adaptation, suggesting that targeting lipid-driven immunometabolic axes could potentiate immunotherapy Pascual and Benitah. Complementing these findings, Zhang et al. investigate the prognostic significance of lipid-metabolism–related gene expression within the tumor immune microenvironment (TIME) of clear cell renal cell carcinoma (ccRCC). Using TCGA and E-MTAB-1980 datasets, the authors identified differentially expressed genes involved in lipid metabolism and overall survival, ultimately constructing an eight-gene prognostic risk model (*ALOX5, DPEP1, HADH, PLIN2, SCD5, SLC44A4, TRIB3, UGT8)*. This model effectively stratified patients into high- and low-risk groups with significantly different survival outcomes and demonstrated robust predictive performance across training, validation, and external cohorts. High-risk patients exhibited lower tumor purity, elevated stromal and immune scores, and higher infiltration of multiple immune cell populations, suggesting that dysregulated lipid metabolism contributes to an altered and more immunologically active TIME associated with poorer prognosis. Functional enrichment analyses further linked the high-risk signature to disturbances in fatty acid metabolism, peroxisomal processes, and immune-response pathways. Among the eight genes, ALOX5 emerged as a key driver and was experimentally validated. Its knockdown reduced lipid accumulation, suppressed proliferation, migration, and invasion of ccRCC cells *in vitro*, and impaired tumor growth *in vivo*, supporting its role as a potential therapeutic target. Overall, the study provides a comprehensive lipid-metabolism-based prognostic model and highlights the mechanistic interplay between metabolic reprogramming and immune microenvironment remodeling in ccRCC, offering new avenues for individualized treatment strategies Zhang et al.

Expanding beyond the immediate context of tumor cell biology, Liu et al. present a timely synthesis exploring how structured exercise modulates systemic lipid metabolism in ways that may restrict tumor metabolic plasticity. Their review provides detailed evidence that aerobic and resistance exercise increase mitochondrial oxidative capacity, enhance fatty acid β-oxidation, and lower circulating lipid substrates, thus competing with tumor cells for essential metabolic resources. Exercise-induced activation of AMPK, downregulation of ACC and FASN, and modulation of CPT1-dependent fatty acid entry into mitochondria collectively reorient systemic metabolism toward a state that is less permissive to tumor growth. Notably, endocrine-responsive tumors such as breast and prostate cancers, which rely heavily on lipid-driven metabolic programs, may be particularly vulnerable to these systemic metabolic shifts. The authors also emphasize the potential of exercise to modulate endocrine axes, including insulin signaling, glucocorticoid responses, and adipokine secretion, thereby indirectly influencing tumor biology and treatment response Liu et al.

Song et al. present a systematic review and meta-analysis evaluating the prognostic significance of circulating lipid levels, including HDL-C, LDL-C, triglycerides, and total cholesterol, in patients with primary breast cancer. Following PRISMA guidelines and PROSPERO registration (CRD42021297118), the authors screened 13,292 records, ultimately including eight cohort studies encompassing 7,186 patients. The meta-analytic findings indicate that lower HDL-C levels show a consistent trend toward poorer disease-free survival (DFS) and overall survival (OS), but pooled hazard ratios did not reach statistical significance (DFS: HR = 1.05, 95% CI 0.87–1.28; OS: HR = 1.10, 95% CI 0.83–1.47). The aggregated data for LDL-C and triglycerides similarly demonstrated no significant associations with DFS or OS, despite heterogeneity across individual studies, suggesting potential subtype-dependent effects, particularly within triple-negative breast cancer (TNBC). Evidence for total cholesterol was limited and inconclusive. The review highlights substantial methodological variability across studies, including inconsistent dyslipidemia thresholds, population homogeneity (predominantly Chinese cohorts), and a lack of stratification by molecular subtype. These limitations constrain generalizability and obscure potential biologically relevant associations between lipid homeostasis and breast cancer progression. Song et al. conclude that while reduced HDL-C may correlate with adverse outcomes, current evidence does not support lipid fractions as robust independent prognostic markers in breast cancer. The authors emphasize the need for multicenter, molecularly stratified cohorts and standardized lipid assessments to clarify whether lipid dysregulation contributes to subtype-specific disease trajectories or treatment responsiveness Song et al.

Kim et al. conducted an extensive retrospective cohort study (*n* = 42.539) to evaluate whether intra-individual lipid variability predicts mortality in newly diagnosed cancer patients not receiving lipid-lowering therapy. Lipid variability quantified as the coefficient of variation (CV) of ≥3 measurements within 2 years of diagnosis was analyzed for total cholesterol (TC), low-density lipoprotein cholesterol (LDL-C), high-density lipoprotein cholesterol (HDL-C), and triglycerides (TG) using multivariable Cox proportional hazards models. Higher TC variability demonstrated a strong dose–response association with all-cause mortality, with adjusted hazard ratios increasing from 1.32 in Q2 to 1.96 in Q4, relative to Q1. LDL-C variability showed a similar but weaker graded association, whereas HDL-C variability was significant only in the highest quartile. TG variability exhibited no association with mortality. Sensitivity analyses censoring at NED status and censoring for initiation of lipid-lowering therapy yielded consistent results. The authors suggest that lipid variability may reflect underlying endothelial dysfunction, vascular instability, or perturbed cholesterol homeostasis, mechanisms that may exacerbate cancer progression or systemic frailty. Given the routine Research Topic of lipid profiles in oncology, lipid variability may serve as a readily accessible prognostic biomarker in cancer patients not exposed to lipid-lowering agents Kim et al.

Collectively, these studies reinforce the concept that lipid metabolism serves as a cross-systemic nexus linking endocrine balance, immune function, and oncogenesis. They highlight the potential of integrating metabolic modulation through lifestyle interventions, pharmacologic lipid metabolism inhibitors, or immune-metabolic reprogramming into comprehensive cancer management. We believe that *Frontiers in Medicine*, with its broad translational scope, provides an ideal platform to foster interdisciplinary discourse on lipid metabolism as a unifying target across organ systems and clinical contexts. Future research should prioritize integrative strategies that combine metabolic exercise paradigms, lipidomic biomarkers, and immune-metabolic therapies to enhance precision oncology.

